# A partial molar pregnancy associated with a fetus with intrauterine growth restriction delivered at 31 weeks: a case report

**DOI:** 10.1186/s13256-019-2150-4

**Published:** 2019-07-04

**Authors:** Pasquale De Franciscis, Antonio Schiattarella, Domenico Labriola, Carolina Tammaro, Enrico Michelino Messalli, Elvira La Mantia, Marco Montella, Marco Torella

**Affiliations:** 1 Department of Woman, Child and General and Specialized Surgery, University of Campania “Luigi Vanvitelli”, Largo Madonna delle Grazie 1, 80138 Naples, Italy; 2 Pathology Unit, Department of Mental and Physical Health and Preventive Medicine, University of Campania “Luigi Vanvitelli”, Naples, Italy

**Keywords:** IUGR, Partial molar pregnancy, Gestational trophoblastic disease, Placental histologic examination, Misdiagnosis

## Abstract

**Introduction:**

Molar pregnancies belong to a group of diseases classified as gestational trophoblastic diseases, which result from an altered fertilization. Partial molar pregnancy with a live fetus is a very rare condition, occurring in 0.005 to 0.01% of all pregnancies; it presents a challenging diagnosis, especially when clinical signs are almost completely absent.

**Case presentation:**

Here we report a rare case of partial molar pregnancy in which a normal-appearing male fetus with diploid karyotype was delivered at 31 weeks gestation by a 37-year-old white woman. The pregnancy was characterized by an episode of threatened abortion in the first trimester and an ultrasonographic diagnosis of intrauterine growth restriction. Our patient did not report any suspicious symptoms for trophoblastic disease. Due to impaired umbilical artery velocimetry with an absence of the diastolic phase, she underwent an emergency caesarean section at 31 weeks and delivered an 880 g male baby. The male baby was normal without any complications at 3-month and 12-month follow-up and the mother had no evidence of recurrence after 3 and 12 months of follow-up. Pathological examination of the placenta showed changes of partial hydatidiform mole.

**Conclusion:**

Partial molar pregnancy with a live fetus is a very rare condition that presents a challenging diagnosis. Recognizing it is of primary importance for patient care and the placenta should always be investigated at birth, especially in a newborn with intrauterine growth restriction.

## Introduction

Hydatidiform mole belongs to a group of diseases classified as gestational trophoblastic disease, which results from an impaired fertilization [1, 2]. Moles have been divided into complete or partial hydatidiform moles on the basis of distinctive histopathological features and genetic abnormalities [3]. Partial molar pregnancy with coexisting fetus is a rare complication with an incidence of 0.005–0.01% of all the pregnancies [1]. It usually derives from dispermic fertilization of a haploid normal oocyte and produces a triploid set of chromosomes (69 XXX, 69 XXY, or 69 XYY) and is most commonly associated with the presence of a malformed fetus [1, 2]. The most relevant symptom of a molar pregnancy is heavy bleeding from the vagina early in the pregnancy. Other symptoms can be severe nausea, hyperemesis, hyperthyroidism, hypertension, and proteinuria, and the occurrence of fetal anemia. Human chorionic gonadotropin (hCG) levels are generally lower than in complete molar pregnancy [3, 4]. A diagnosis can be achieved with ultrasonography and sensitive measurement of serum hCG, usually on first trimester. However, partial moles are often misdiagnosed as an incomplete or missed abortion of the first trimester. In less than 25% of cases, pregnancy with a partial mole and a single normal fetus evolves to a viable fetus: in such cases the diagnosis can be suspected or misunderstood [5] and the management is a challenge for potential maternal and fetal complications. Here we report a rare case of misdiagnosed partial molar pregnancy in which a normal-appearing male fetus with diploid karyotype was delivered at 31 weeks of gestation.

## Case presentation

Our patient was informed of all procedures she was to undergo; she signed a consent allowing data collection for research purposes, and gave full approval for the report and publishing of the case. This case is in accordance with the Declaration of Helsinki, in accordance with the Consensus-based Clinical Case Reporting Guideline Development (http://www.equator-network.org/), and the Committee on Publication Ethics (COPE) guidelines (http://publicationethics.org/), and approved by the Institutional Review Board (IRB) of the university hospital in which it was reported [6].

**Fig. 1:**
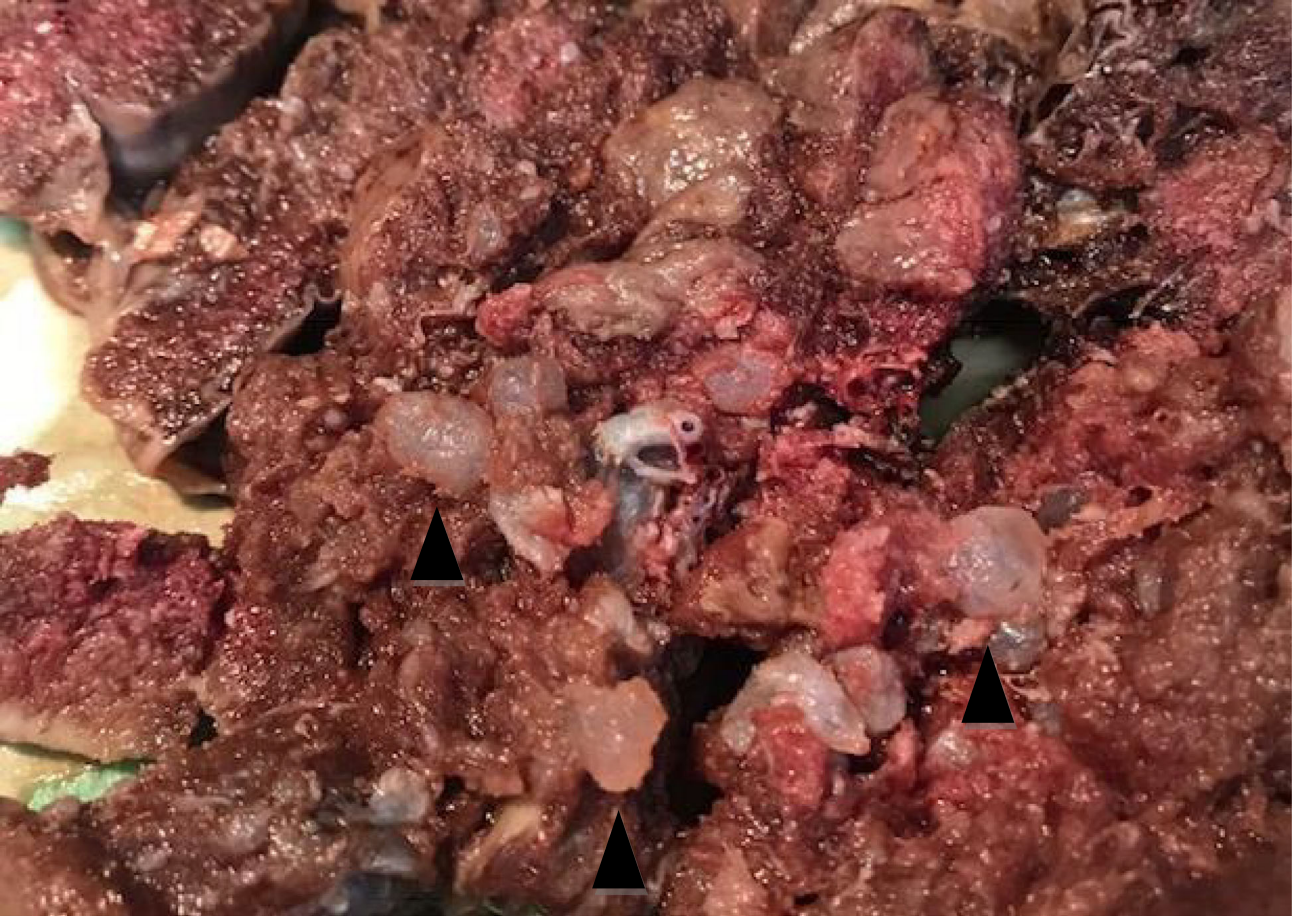
Hydatidiform molar villi. Note the bulbous swelling of terminal villi and the slender nature of main stem villi (*black arrowhead*)

A 37-year-old white woman, gravida 1, at 30 weeks and 5 days of gestation was admitted in March 2018 to the Department of Woman, Child and General and Specialized Surgery of University of Campania “Luigi Vanvitelli” (Naples, Italy) with suspected fetal restricted growth. She had already attended the out-patient fertility center of our Department for a path of infertility, with the execution of genetic, laboratory, and antibody tests that gave a negative result. Subsequently, a spontaneous pregnancy occurred. Her obstetric history was characterized by an episode of threatened abortion in the first trimester. Serum titers of b-hCG were 7276 mIU/mL and 14,898 mIU/mL at 5 weeks and 1 day and 5 weeks and 3 days of gestation, respectively. An ultrasound examination at 5 weeks and 5 days revealed an empty gestational sac with no findings suspect for gestational trophoblastic disease. The following ultrasound examinations showed a regular ongoing pregnancy with a single fetus. A structural ultrasound examination at 20 weeks showed an altered flow rate of uterine artery which justified the use of daily low-dose aspirin (100 mg) for prophylaxis of preeclampsia. Thyroid-stimulating hormone (TSH) levels in the first and second trimester were in the normal range. A fetal echocardiography was performed at 24 weeks because non-invasive first-trimester screening for chromosomal abnormalities was not achieved: the result was normal. Our patient was admitted to our department due to worsening of the biometric and Doppler velocimetry parameters, at 30 weeks and 5 days of gestation. An admission ultrasound examination revealed a singleton pregnancy with no fetal structural abnormalities, but fetal biometry was not consistent with gestation with an estimated weight of 1000 g, below the third percentile for gestational age (consistent with 27 weeks). Therefore, a symmetrical intrauterine growth restriction (IUGR) was diagnosed. Amniotic fluid was regular in quantity and the placenta appeared regular. Umbilical artery Doppler velocimetry was impaired due to the absence of the diastolic phase. Our patient was given two injections of 12 mg of betamethasone 24 hours apart to prevent respiratory distress syndrome and she underwent an emergency cesarean section at 31 weeks and 1 day. A phenotypically normal alive and healthy male infant weighing 880 g was delivered. Macroscopic evaluation of the placenta showed a 14 × 10 × 3.5 cm discoid placenta with a weight of 416 g. On the maternal surface of the placenta, the presence of focal “grape” vesicles were observed, which occupied approximately 5% of the peripheral surface (Fig. 1).

**Fig. 2:**
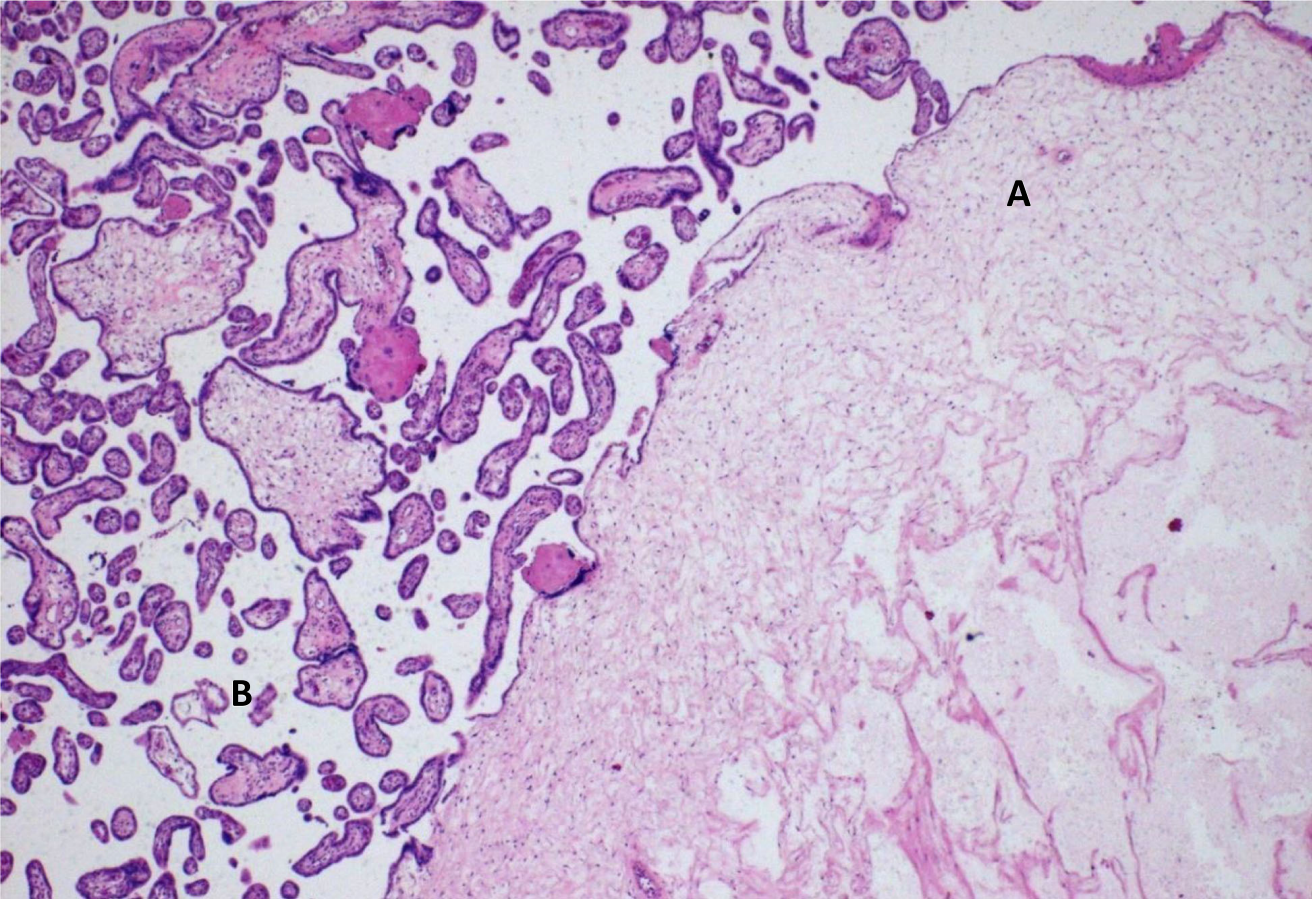
Focal intermingling of partial molar (*A*) with normal villi (*B*). Hematoxylin and eosin, × 400

**Fig. 3:**
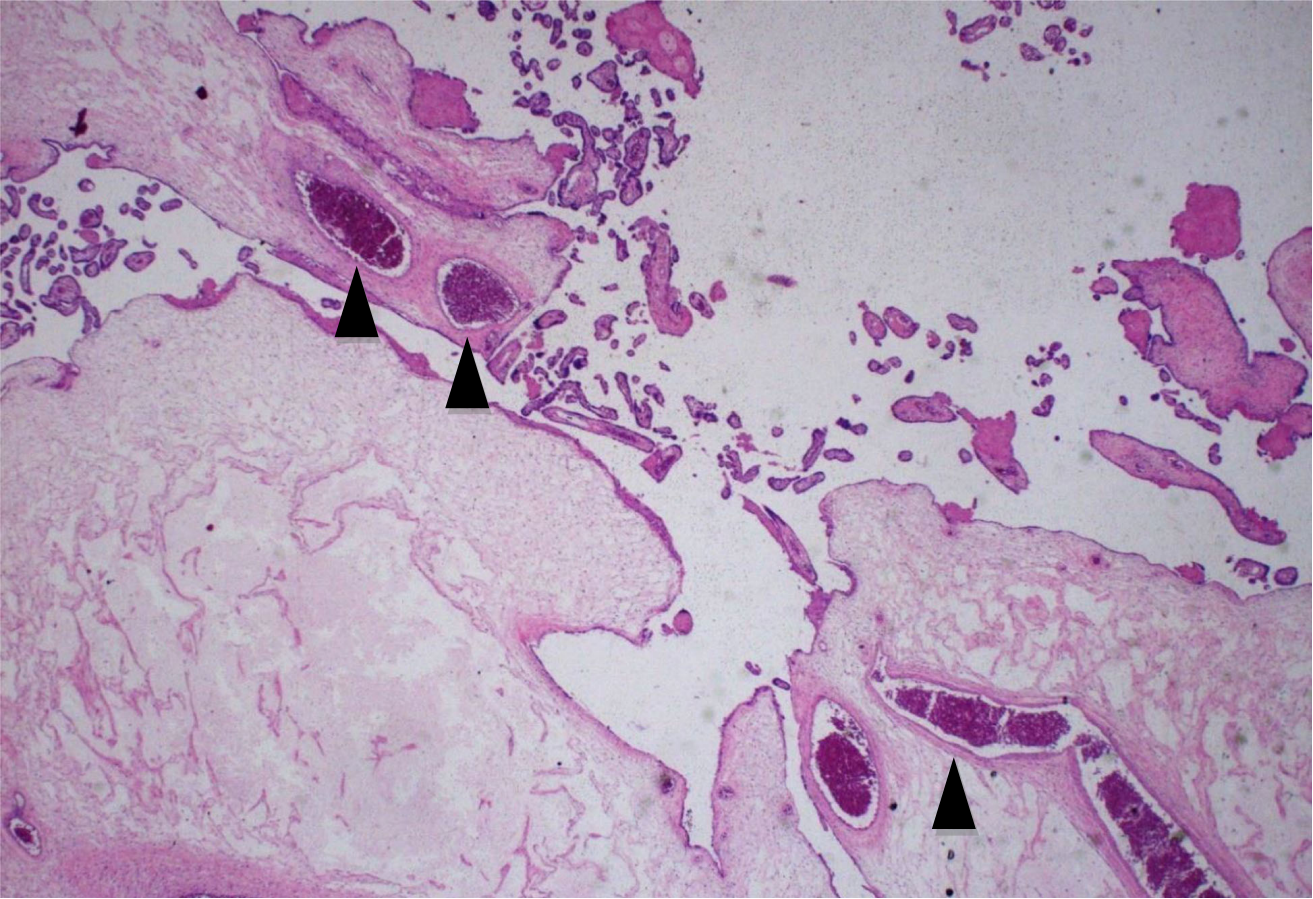
In this microscopic image is observed partial molar villus with intact perfused fetal blood vessel (*black arrowheads*). Hematoxylin and eosin, × 400

On microscopic examination, these “grape-like areas” showed large-sized and medium-sized chorionic villi, with a festooned pattern, and numerous gross nodular swellings culminating in a “cistern-like” formation in the stromal axis and variable proliferation of trophoblast (Fig. 2) with normal blood vessels (Fig. 3).

The other 95% of the placenta was composed of small, hypercapillarized chorionic villi (terminal villi). The pathological diagnosis was placenta at third trimester with associated partial mole modifications. Our patient was in good health and had no evidence of recurrence after 3 and 12 months of follow-up. The male baby was normal and the result of a karyotype analysis was diploid. No complications occurred at 3-month and 6-month follow-up and the baby is still living. After delivery, hCG level was negative (less than 5 mIU/mL) and the mother had 12 consecutive months of negative hCG levels and no metastases were found.

## Discussion

Partial molar pregnancy with coexisting fetus is an extremely rare variation of a molar pregnancy: it accounts for 0.005 to 0.01% of all pregnancies and usually derives from dispermic fertilization of a haploid normal oocyte and produces a triploid set of chromosomes [1, 7]. An increased incidence could be explained by the greater use of assistive reproductive techniques [8]. There are three types of molar pregnancy with coexisting normal live fetus: the most frequent case is a twin pregnancy with one normal fetus having a normal placenta and another complete mole; the second type is a twin pregnancy with regular fetus and placenta and another partial mole [9]; the third and most uncommon occurrence reported only 19 times in the literature is a singleton normal fetus with partial molar placenta, which is similar to our case [5]. The diagnosis of molar pregnancy with coexisting fetus is difficult. In fact, the diagnosis can be achieved with ultrasonography and sensitive measurement of serum hCG, usually on first trimester [10]. Moreover, in molar pregnancy, symptoms are hyperemesis, heavy bleeding from the vagina, increased blood pressure and, sometimes, proteinuria that can lead to preeclampsia [7, 10, 11]. Sometimes women with hydatidiform mole may experience symptoms typical of hyperthyroidism because of extremely high levels of hCG; in fact, this hormone can mimic the action of TSH [12, 13]. High concentrations of hCG and suppressed levels of TSH can help to confirm the diagnosis. Interruption of pregnancy is common owing to congenital anomalies like triploidy of the fetuses and severe intrauterine fetal growth restriction due to limited normal functional placental circulation. If the pregnancy does not stop, management of molar pregnancies with an apparently normal fetus remains challenging [5, 14]. The woman must be counseled regarding the maternal and fetal complications: late abortion, vaginal bleeding, mal presentations, preterm labor, persistent gestational trophoblastic disease, severe anemia in the fetus, hyperthyroidism, hypertensive disorders of pregnancy, pulmonary edema, and thromboembolic phenomena [15, 16]. Therefore, pregnancy needs to be followed with regular ultrasound assessment of fetal anatomy and growth. A chorionic villous biopsy could be done if a live fetus is present, to confirm the diagnosis and to differentiate between a partial mole and complete mole: it has been reported that the latter has approximately 20% tendency to become an invasive mole or even a choriocarcinoma, while the risk was lower for partial moles [15, 17].

## Conclusion

In our case, the diagnosis was not suspected and only the anomalous presence of IUGR could be interpreted as a sign of a placental defect and, among the various causes, could be referred to as a molar pregnancy. As a consequence, histological examination of the placenta is essential in all cases of IUGR. After the delivery, it is mandatory to check the patient until there have been 12 consecutive months of negative hCG levels. In conclusion, in every case of molar pregnancy suspect for a normal fetus, adequate counseling of the patient and a strict follow-up during and after pregnancy for the risk of persistence of the trophoblastic pathology are necessary [18–20].

## Data Availability

The dataset used and/or analyzed during the current study is available from the corresponding author on reasonable request.
